# Hyperleukocytosis-induced stroke and tonsillar herniation: Case report

**DOI:** 10.1016/j.amsu.2021.102776

**Published:** 2021-09-03

**Authors:** Hasan Hashem, Baha'eddin A. Muhsen

**Affiliations:** a Department of Pediatrics, Division of Pediatric Hematology Oncology and Bone Marrow Transplantation, King Hussein Cancer Center, Amman, Jordan; b Department of Surgery, Division of Neurosurgery, King Hussein Cancer Center, Amman, Jordan

**Keywords:** Ischemic stroke, Tonsillar herniation, Hyperleukocytosis, Hyperviscosity, Leukemia

## Abstract

**Introduction:**

and Importance: Acute ischemic stroke is a rare event in children with leukemia, yet with long-term morbidity, substantial health, and economic cost. Central nervous system (CNS) leukemic involvement and chemotherapy-related stroke are the most common causes. Hyperleukocytosis induced stroke is very rarely reported.

**Case presentation:**

A 2-year-old male child presented with hyperleukocytosis (leukocyte count was 320x10^9^/L). Bone marrow evaluation revealed T-cell acute lymphoblastic leukemia. He was treated with dexamethasone, vincristine and daunorubicin, and on day 4 of chemotherapy, he developed abnormal movements, altered mental status, limb weakness and mutism. Magnetic resonance imaging of the brain showed multifocal infarctions involving left pons and both cerebellar hemispheres causing tonsillar herniation with restricted diffusion and mild hydrocephalus but no leptomeningeal enhancement or leukemic infiltrates. Magnetic resonance angiography did not show any arterial stenosis. He was intubated, sedated and managed conservatively with dexamethasone. Cytologic analysis of cerebrospinal fluid showed no blasts. Thrombophilia work up was negative. Five weeks later, the patient had significant improvement in overall neurologic status. He is free of leukemia. MRI showed interval resolution of previous infarcts.

**Clinical discussion:**

Hyperviscosity secondary to hyperleukocytosis was considered to be the most likely explanation for this patient stroke after excluding thrombophilia and leukemic infiltration. Prompt management with hydration and careful chemotherapy resulted in good outcome in our patient.

**Conclusion:**

This case demonstrate the value of early recognition and prompt management of posterior circulation ischemic stroke in children with leukemia and hyperleukocytosis at presentation.

## Highlights

• Hyperleukocytosis and hyperviscosity can result in arterial ischemic stroke
• Posterior circulation ischemic strokes are very rare in children with leukemia
• Posterior circulation stroke can carries potential risk of herniation
• Prompt recognition and management are essential in posterior circulation stroke

## 1 Introduction

Acute ischemic stroke is a rare event in children, yet with long-term morbidity and substantial health, and economic cost. In addition to vasculopathy, thrombophilia, head trauma, and cardiac disorders as risk factors for ischemic stroke, cancer and chemotherapy treatment are also known predisposing factors [1]. Hematologic malignancies, such as acute leukemia, in particular can cause both hemorrhagic and thrombotic complications in both venous and arterial vessels [2,3].

There are a number of problems associated with the central nervous system (CNS), with the leukemia itself and with its treatment. One of the most common complications related to CNS is leukemic infiltration, which has been analyzed extensively. However, CNS complications excluding leukemic involvement have rarely been reported [[5], [6], [7]]. Arterial ischemic strokes related to hyperleukocytosis are rarely observed. We report herein a young child who developed multiple posterior circulation ischemic strokes and tonsillar herniation at the time of presentation of his leukemia. This case report has been reported according to SCARE guidelines [4].

## 2 Case Presentation

A 2-year-old male child presented to emergency department by his parents' car with hyperleukocytosis (leukocyte count was 320 x 10^9^/L). Bone marrow aspirate and biopsy confirmed the diagnosis of T-cell acute lymphoblastic leukemia. The patient was admitted to pediatric intensive care unit due to his elevated leukocyte count and managed with hydration and allopurinol as tumor lysis precaution. The next day, the patient was started on chemotherapy. He received one dose of intravenous vincristine (1.5 mg/m^2^) and daunorubicin (25 mg/m^2^) on day 1, and started on dexamethasone. The patient's leukocyte count dropped to 5.2 x 10^9^/L on day 3 of chemotherapy.

**Fig. 1:**
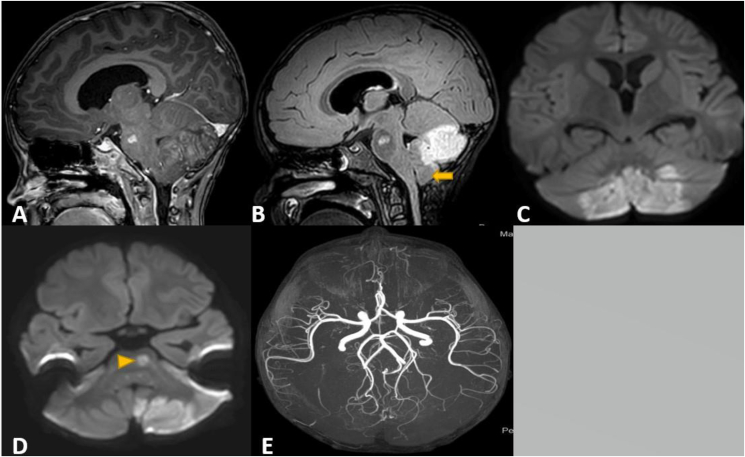
Magnetic resonance imaging of the brain (MRI). A: Sagittal view with contrast showing cerebellar gyri and pontine enhancement with mild tonsillar herniation, B: Flair sequence showing cerebellar edema, mild hydrocephalus and 3 mm tonsillar herniation (arrow), C–D: Diffusion-Weighted view showing avid restriction involving pons (arrowhead) and the medial inferior aspect of both cerebellar hemispheres confined to the territory of the medial branches of the posterior inferior cerebellar arteries, E: Magnetic resonance angiography (MRA) sequence shows no evidence of arterial stenosis.

The next day (day 4 of chemotherapy), the patient developed abnormal movements, altered mental status, limb weakness and mutism. Magnetic resonance imaging of the brain (MRI) showed multifocal infarctions involving left pons and inferomedial aspect of both cerebellar hemispheres causing mild tonsillar herniation (Fig. 1A and B) with restricted diffusion (Fig. 1C and D) and mild hydrocephalus but no leptomeningeal enhancement or leukemic infiltrates. Magnetic resonance angiography (MRA) was normal and internal carotid and vertebrobasilar arteries were unremarkable for stenosis or obstruction, circle of Willis with no stenosis or aneurysmal dilatation (Fig. 1E). The patient was intubated, sedated and managed conservatively with dexamethasone. Asparaginase was not started due to the recent stroke. No anticoagulation was used due to high bleeding risk secondary to thrombocytopenia and to avoid hemorrhagic transformation of his stroke. Cytologic analysis of cerebrospinal fluid showed no blasts. Family history was negative for thrombophilia. Thrombophilia work up was negative including protein C, protein S, lupus anticoagulant, beta 2 glycoprotein and anticardiolipin antibodies. Prothrombin gene mutation and factor V Leiden gene mutations were both normal. Disease evaluation on day 8 of induction chemotherapy revealed no leukemic blasts in his peripheral blood. Flow cytometry analysis revealed negative minimal residual disease. Hyperviscosity secondary to hyperleukocytosis was considered to be the most likely explanation for his arterial stroke.

**Fig. 2:**
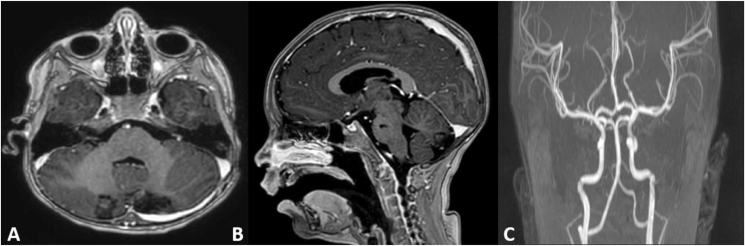
MRI brain with contrast 8 weeks follow-up showed interval resolution of previous infarcts. A: Axial view, B: sagittal view, C: Magnetic resonance angiography (MRA) showing no evidence of arterial stenosis.

Five weeks later, the patient had significant improvement in overall neurologic status. BM evaluation showed no blasts and flow cytometry analysis for minimal residual disease was negative. Repeat brain MRI at 8 weeks from the insult showed interval resolution of previous infarcts (Fig. 2). The patient is currently 6 months throughout treatment receiving maintenance chemotherapy and is compliant.

## 3 Discussion

Acute ischemic stroke is rare in children and is less frequent in the posterior circulation compared to anterior circulation [1]. Moreover, early acute ischemic stroke is a very rare and potentially life-threatening complication in children with leukemia at time of presentation [2,3]. Hyperleukocytosis leading to hyperviscosity is likely the cause for our patient's ischemic stroke. Patients with posterior circulation ischemic stroke often deteriorate quickly due to rapid increase in intracranial pressure and potential risk for herniation [8,9].

In our case, conservative management with dexamethasone and systemic chemotherapy without asparaginase appears to be the treatment of choice. Suboccipital craniectomy as therapy for herniation or external ventricular drain for hydrocephalus might be a possible therapeutic options. Goeggel-Simonetti and colleagues showed that neurosurgical procedures, were only performed in 9.1% of children with posterior circulation ischemic strokes [1]. However, it remains challenging in children with leukemia due to high bleeding risk secondary to low platelet count. Moreover, no anticoagulation was used in our patient due to high bleeding risk secondary to thrombocytopenia and to avoid hemorrhagic transformation. In addition, the standard treatments for ischemic stroke might not be effective due to the leukocyte-rich nature of thrombi related to hyperviscosity related to hyperleukocytosis. Our study calls for both prompt parenchymal and vascular imaging and close observation and prompt management with hydration and careful chemotherapy for children with leukemia and posterior circulation ischemic stroke.

## 4 Conclusion

Hyperleukocytosis and resultant hyperviscosity can result in arterial ischemic stroke in young children with leukemia. Posterior circulation ischemic strokes are very rare in children with leukemia especially at time of leukemia diagnosis and patients can deteriorate quickly due to rapid increase in intracranial pressure and potential risk of herniation. Prompt recognition and management with hydration and careful chemotherapy are essential in children with leukemia and posterior circulation stroke.

## Consent

Written informed consent was obtained from the patient's family to publish this case report and accompanying images. A copy of the consent is available for review by the Editor-in-Chief of this journal on request.

## Conflict of interest statement

The authors have no competing conflict of interest.

## Source of funding

None.

## Ethical approval

Institutional review Board of King Hussein Cancer Center approved this case report.

## Research registration

Not applicable.

## Provenance and peer review

Not commissioned, externally peer reviewed.

## Author contribution

HH and BM both wrote the manuscript, collected the data, analyzed the images, and approved the final version of this manuscript.

## Guarantor

Hasan Hashem, MD, Department of Pediatrics, Division of Pediatric Hematology Oncology and Bone Marrow Transplantation, King Hussein Cancer Center, 202 Queen Rania street, Amman, Jordan 11941. Fax: +962–6(5353001). Phone: +962–6(5300460). Email: HH.08847@khcc.jo.

## Declaration of competing interest

The authors have no competing conflict of interest.

## Registration of research studies

Name of the registry: NA.

Unique Identifying number or registration ID: NA.

Hyperlink to your specific registration (must be publicly accessible and will be checked): NA.
